# ΔNp63 to TAp63 expression ratio as a potential molecular marker for cervical cancer prognosis

**DOI:** 10.1371/journal.pone.0214867

**Published:** 2019-04-11

**Authors:** Sunyoung Park, Suji Lee, Jungho Kim, Geehyuk Kim, Kwang Hwa Park, Tae Ue Kim, Dawn Chung, Hyeyoung Lee

**Affiliations:** 1 Department of Biomedical Laboratory Science, Yonsei University, Wonju-si, Gangwon-do, Republic of Korea; 2 Department of Pathology, Yonsei University Wonju College of Medicine, Wonju-si, Gangwon-do, Republic of Korea; 3 Department of Obstetrics and Gynecology, Gangnam Severance Hospital, Yonsei University College of Medicine, Seoul, Republic of Korea; Sapporo Ika Daigaku, JAPAN

## Abstract

p63 is a transcription factor p53 family. Two major isoforms of p63, TAp63 with transactivation (TA) domain and ΔNp63 with truncated TA domain, have been reported to play opposing roles either in tumor suppression or oncogenic function. Little is known about the association of these two isoforms of p63 in the carcinogenesis of cervical cancer. In this study, the mRNA expression levels of TAp63 and ΔNp63 in 40 normal, 30 low-grade squamous intraepithelial lesions (LSIL), 38 high-grade squamous intraepithelial lesions (HSIL), and 52 cervical cancer formalin-fixed paraffin-embedded tissues were examined using quantitative reverse transcription polymerase chain reaction (RT-qPCR). We analyzed the association between the ΔNp63 and ΔN/TAp63 mRNA expression ratio and clinicopathological parameters and compared disease-specific survival of each ΔNp63 mRNA expression and ΔN/TAp63 mRNA expression ratio. The ΔN/TAp63 mRNA expression ratio in cervical cancer showed higher sensitivity than the mRNA expression levels of ΔNp63 (52.0% vs 44.2%). The level of ΔN/TAp63 mRNA expression ratio in precancerous LSIL and HSIL was higher than in normal tissues (*P* = 0.01 and *P* = 0.003) and lower than in cervical cancer tissues (*P* = 0.03 and *P* = 0.02). Besides, the positive ΔN/TAp63 mRNA expression ratio was associated with bulky tumor size and high expression of Ki-67, the proliferation marker, in cervical cancer (*P* = 0.04 and *P* = 0.02). The cervical cancer patients with the positive ΔN/TAp63 mRNA expression ratio showed worse survival compared to those who with the negative expression ratio of ΔN/TAp63 (HR = 5.7, 95% CI: 1.6–19.9). In conclusion, the balance of TAp63 and ΔNp63 is closely related to the carcinogenesis of cervical cancer. The ΔN/TAp63 mRNA expression ratio could be useful as a diagnostic and prognostic marker of cervical cancer.

## Introduction

Cervical cancer is one of the leading causes of mortality and is ranked as the third most common cancer in women worldwide [1]. According to the World Health Organization, cervical cancer accounts for approximately 530,000 new cases and 266,000 deaths per year worldwide [2]. Annually, approximately 3,500 patients are diagnosed with and 960 patients die from cervical cancer in Korea [3].

p63 and p73 are members of the p53 family, which is a well-known tumor suppressor gene, because of their structural and functional similarities [4]. The p53 family consists of a transactivation (TA) domain, a DNA-binding domain, and an oligomerization domain. Generally, these proteins induce cell cycle arrest and apoptosis [4–6]. p63 encodes for two isoforms, TAp63 and ΔNp63, by alternative splicing of two promoters (P1 and P2). P1 leads to the TAp63 isoform which contains a TA domain at the N-terminus, and P2 synthesizes the ΔNp63 isoform, which lacks the TA domain [5, 7].

p63 proteins function in epithelial stratification, differentiation, and proliferation of epithelial stems cells [8, 9]. p63 is related to normal development and homeostasis, as well as various types of cancer, such as squamous cell carcinoma (SCC) of the head and neck, cervix, and lungs and adenocarcinoma (ADC) of breast [10–13]. Specifically, TAp63 has a tumor suppressive function to maintain epithelial stem cells and induce cell cycle arrest, senescence, and apoptosis, while ΔNp63 has an oncogenic function in the differentiation of embryonic cells to epidermal cells, cell proliferation, self-renewal, and inhibition of senescence and apoptosis [5].

Billant et al. suggested that ΔNp63 acts as a dominant-negative inhibitor of TAp63 [14]. Two models for this dominant-negative action were recently reported. First, ΔNp63 binds to the target gene promoter and prevents TAp63 from binding. The second model involves the oligomerization domain found in each member of the p53 family. To bind p63 to DNA, tetramers must be formed. When TAp63 and ΔNp63 form a heterotetramer, transcription activity decreases compared to a TA homotetramer [6, 15]. Later, both TAp63 and ΔNp63 transcripts undergo C-terminal alternative splicing to yield six further C-terminal isotypes, TAp63α, β, γ, and ΔNp63α, β, γ [5, 6, 16]. Because these two isoforms of p63 (TAp63 and ΔNp63) show paradoxical roles and closely related expression, the differences in ΔNp63 expression and the ΔNp63 to TAp63 (ΔN/TAp63) expression ratio in cervical cancer and precancerous lesions (low-grade squamous intraepithelial lesions [LSIL] and high-grade squamous intraepithelial lesions [HSIL]) should be examined.

The aim of this study was to examine the mRNA expression levels of TAp63 and ΔNp63 in cervical formalin-fixed paraffin-embedded (FFPE) tissues. Each isoform and the ΔN/TAp63 expression ratio were investigated to determine their potential as diagnostic and prognostic markers. Finally, the relationships between ΔNp63 and the ΔN/TAp63 expression ratio with clinicopathological parameters such as tumor size, stage, lymph nodes, and human papillomavirus (HPV) test and prognosis were determined.

## Materials and methods

### Cell culture

SiHa, HeLa, C33A, CaSki, and ME-180 cells were purchased from American Type Culture Collection (ATCC, Manassas, VA, USA) and Korean Cell Line Bank (Seoul, Korea). SiHa cells and HeLa were cultured in Dulbecco’s Minimal Essential Medium containing 10% fetal bovine serum (FBS) and penicillin-streptomycin. C33A cells were cultured in Minimal Essential Medium containing 10% FBS and penicillin-streptomycin. Caski and ME180 cells were cultured in Roswell Park Memorial Institute medium containing 10% FBS and penicillin-streptomycin. Cells counted using a T20TM Automated cell counter (Bio-Rad, Hercules, CA, USA) according to the manufacturer’s instructions.

Five cervical cancer cell lines with different characteristics were used: SiHa, C33A, CaSki, and ME-180 were SCC and HeLa was ADC. Additionally, the cells show different HPV types: ME-180 is HPV39-positive, SiHa and CaSki are HPV-16-positive, HeLa is HPV-18-positive, and C33A is HPV-negative [17].

### Total RNA extraction from cell lines

To extract RNA in cervical cancer cell lines, total cellular RNA was isolated using Isol-RNA Lysis Reagent (5 Prime, Austin, TX, USA) according to the manufacturer’s instructions. The RNA pellet was eluted in 30 μL of diethylpyrocarbonate (DEPC)-treated water (Intron Biotechnology, Seoul, Republic of Korea). The purity and concentration of total RNA were determined by measuring the absorbance at 260 and 280 nm using the Infinite 200 (Tecan, Männedorf, Switzerland). All steps in the preparation and handling of total RNA were performed in a laminar flow hood under RNase-free conditions. The isolated total RNA was stored at −70°C until use.

### Ethics statement

This study was approved by the Institutional Ethics Committee at Yonsei University Wonju College of Medicine (approval no. CR315052). Informed written consent was obtained from all the study participants. All 160 FFPE tissue samples were collected at the Department of Pathology, Yonsei University Wonju Severance Christian Hospital, Wonju, Republic of Korea, between 2010 and 2013. All data were fully anonymized before access.

### Clinical samples

FFPE tissue samples from cervical cancer patients were obtained at the time of first diagnosis. Total 160 FFPE tissue samples were including 40 (25%) normal cervical tissues, 30 (18.8%) LSILs, 38 (23.7%) HSILs, and 52 (32.5%) cervical cancer tissues. All cervical cancer tissues were histologically diagnosed as SCC. For the cervical cancer cases, clinicopathological parameters such as patients’ age, tumor size, FIGO stage, lymph node metastasis, and HPV test were retrospectively reviewed from the patients’ electrical medical records. Among 52 cervical cancer cases, 22 (42.3%) cases had more than 4 cm of tumor size, 28 (53.9%) cases showed stage IIB or more than IIB, 22 (42.3%) cases had lymph node metastasis, and 44 (84.6%) cases showed HPV positive (Table 1). Disease-specific survival time was defined as the period from the date of the first treatment to the date of cancer-related death.

**Table 1:** Clinicopathological characteristics of 160 FFPE tissue samples.

	Normal (n = 40) n (%)	LSIL (n = 30) n (%)	HSIL (n = 38) n (%)	SCC (n = 52) n (%)
Age				
< 50 years	26 (65.0)	17 (56.6)	31 (81.6)	19 (39.5)
≥ 50 years	14 (35.0)	13 (43.3)	7 (18.4)	33 (63.5)
Tumor size				
< 4 cm				30 (57.7)
≥ 4 cm				22 (42.3)
FIGO Stage^a^				
IA-IIA				21 (40.4)
IIB-IVB				28 (53.9)
Unknown^b^				3 (5.8)
Lymph node metastasis				
Negative				28 (53.8)
Positive				22 (42.3)
Unknown^b^				2 (3.8)
HPV test				
Negative				8 (15.4)
Positive				44 (84.6)

^a^FIGO, International Federation of Gynecology and Obstetrics;

^b^Unknown, no diagnostic records.

### Deparaffinization of FFPE tissue and total RNA extraction

To remove paraffin from FFPE tissue samples, 3–4 pieces of 10-μm-thick sections of cervical FFPE tissues were placed in a 1.5-mL microcentrifuge tube and 320 μL of deparaffinization solution (Qiagen, Hilden, Germany) was added and vortexed, followed by incubation for 3 min at 56°C. For RNA extraction, Qiagen RNeasy FFPE kits (Qiagen) were utilized according to the manufacturer’s protocol and 25 μL of RNA was eluted. The purity and concentration of total RNA were determined by measuring the ratio of the absorbance at 260 and 280 nm with an Infinite 200. All preparation and handling procedures were conducted under RNase-free conditions. Isolated total RNA was stored at -70°C until use.

### cDNA synthesis

cDNA was synthesized using total RNA. Briefly, 0.25 μg of random hexamers (Invitrogen, Carlsbad, CA, USA), dNTP mixture (2.5 mM each), DEPC-treated water, and RNA were mixed. The PCR samples were incubated at 65°C for 5 min and chilled on ice. Next, 5X buffer (Invitrogen), 0.1 M DTT (Invitrogen), and 200 U Murine Molony Leukemia Virus Reverse Transcriptase (MMLV-RT; Invitrogen) were added. cDNA synthesis was performed for 10 min at 25°C, followed by 50 min at 37°C and 15 min at 70°C.

### HPV test

To detect HPV E6/E7 mRNA in FFPE cervical tissues, multiplex RT-qPCR was performed using the TaqMan assay with the OPTIMYGENE HPV E6/E7 mRNA RT-qDx assay kit (Optipharm, Osong, Republic of Korea). PCR primers and the corresponding TaqMan probes can detect 16 high risk HPV (hrHPV) genotypes: HPV 16, 18, 31, 33, 35, 39, 45, 51, 52, 53, 56, 58, 59, 66, 68, and 69.

### Design of optimal p63 primers and probes

To develop TAp63 and ΔNp63 specific oligonucleotide primers and TaqMan probes optimized for the RT-qPCR system, the National Center for Biotechnology Information reference sequence and PrimerQuest software program (Integrated DNA Technologies, Coralville, IA, USA) were used. The reference sequences and target sequences for the primers and probes of TAp63 and ΔNp63 are listed in S1 Table.

### RT-qPCR for TAp63 and ΔNp63 mRNA

TAp63 and ΔNp63 mRNA RT-qPCR were performed using 10 μL of 2x Thunderbird probe qPCR mix (Toyobo, Osaka, Japan), 3 μL of primer and TaqMan probe mixture, 2 μL of template cDNA, and distilled water to a final volume of 20 μL per sample. No-template controls as negative controls were included in each run and contained sterile distilled water rather than template DNA. The PCR cycle was run as follows: 95°C for 3 min, followed by 40 cycles of 95°C for 3 s, and 55°C for 30 s. mRNA levels were quantified by determining the cycle threshold (C_T_), which is defined as the number of PCR cycles required for fluorescence to exceed a value significantly higher than that of the background fluorescence. To avoid false-negatives because of mRNA degradation, glyceraldehyde-3-phosphate dehydrogenase was used as an internal control. The amount of TAp63 and ΔNp63 mRNA was determined using the comparative C_T_ method (ΔΔC_T_ method), measuring mRNA relative to a reference gene using CFX Manager Software v1.6 (Bio-Rad).

### Statistical analysis

Receiver operating characteristic (ROC) curves were used to validate optimal value for differentiating normal from cancer tissues. Student’s *t*-test was utilized to confirm the characteristics of TAp63 and ΔNp63 in cervical cancer cell lines and also compare the expression level of the each cervical tissue sample. To test the balance between TAp63 and ΔNp63, the ratio of p63 was calculated as by ΔNp63 ΔΔC_T_/TAp63 ΔΔC_T_, referred as the ΔN/TAp63 expression ratio. Chi-square test was used to analyze the associations between the positivity of ΔN/TAp63 expression ratio and clinicopathological prognostic parameters. Fisher’s exact test was used for the parameters including fewer than five cases.

The survival was estimated by the Kaplan–Meier method and determined the significant differences by the log-rank test. For all tests, *P-*value < 0.05 was considered to be statistically significant and performed by using GraphPad Prism v5.02 (GraphPad, La Jolla, CA, USA).

## Results

### TAp63 and ΔNp63 mRNA expression levels in cervical cancer cell lines

**Fig 1:**
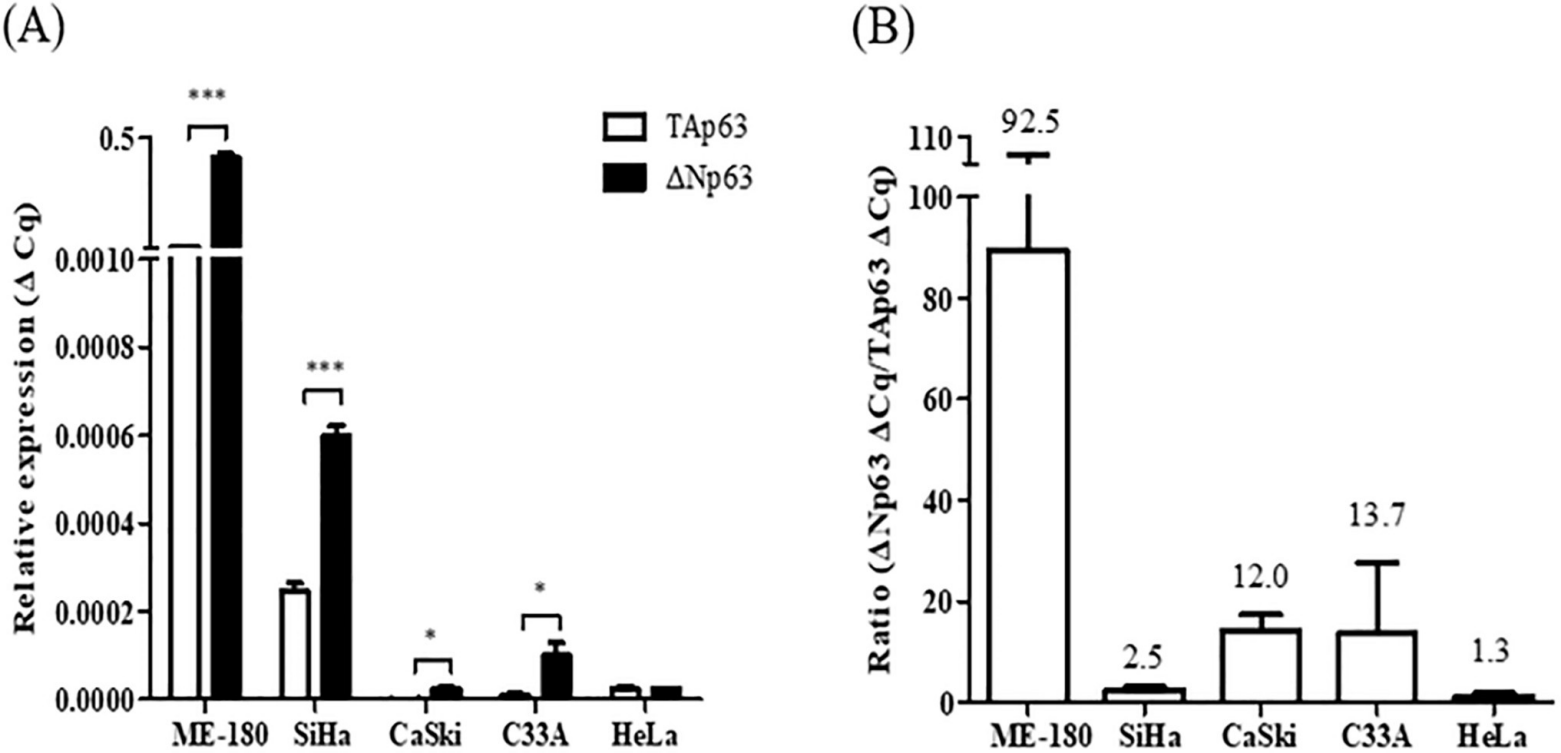
Expression levels of TAp63 and ΔNp63 mRNA and the ΔN/TAp63 mRNA expression ratio in cervical cancer cell lines. (A) TAp63 and ΔNp63 mRNA expression levels in five cervical cancer cell lines including ME-180, SiHa, CaSki, C33A, and HeLa cells were measured by RT-qPCR. (B) The ΔN/TAp63 mRNA expression ratio was analyzed using cervical cancer cell lines. *P < 0.05, ** *P* < 0.01, ***P < 0.001.

The mRNA expression levels of TAp63, characterized by the tumor suppressive function, and ΔNp63, characterized by oncogenic function in cervical cancer, were measured in five cervical cancer cell lines, ME-180, SiHa, CaSki, C33A, and HeLa. To identify whether two isoforms, TAp63 and ΔNp63, made by alternative splicing from p63 were expressed in cervical cancer, the mRNA expression levels of TAp63 and ΔNp63 in the five cervical cancer cell lines were compared. Compared to the mRNA expression levels of TAp63, ΔNp63 mRNA expression levels were significantly higher in the ME-180, SiHa, CaSki, and C33A cell lines (*P* < 0.0001, *P* = 0.0003, *P* = 0.01, and *P* = 0.039, respectively) (Fig 1A). Since ΔNp63 displayed higher mRNA expression levels than TAp63, we investigated whether the disruption of the balance between TAp63 and ΔNp63 could be related to cervical cancer. The ΔN/TAp63 mRNA expression ratio was 92.5-fold in ME-180, 2.5-fold in SiHa, 12.0-fold in CaSki, 13.7-fold in C33A, and 1.3-fold in HeLa cells; this identified the overexpression of the ΔN/TAp63 ratio in all cervical cancer cell lines (Fig 1B).

### TAp63 and ΔNp63 mRNA expression levels in cervical cancer and normal tissues

We then explored the mRNA expression levels of TAp63 and ΔNp63 in 40 normal FFPE tissues and 52 cervical cancer FFPE tissues. The mRNA expression levels of TAp63 showed no significant difference between normal and cancer tissues (*P* = 0.61). However, the mRNA expression levels of ΔNp63 were significantly increased in cancer (*P* = 0.0002). When each TAp63 and ΔNp63 for clinical use were subjected to ROC curve analysis, the AUC value of TAp63 was 0.5135 (95% confidence interval [CI] 0.3891–0.6378) and that of ΔNp63 was 0.7529 (95% CI 0.6549–0.8509). According to the ROC curve analysis, the ΔNp63 cut-off value determined by the best sensitivity and specificity was 7.6. At a cut-off value of 7.6, the sensitivity of ΔNp63 was 44% and the specificity was 95% (S1 Fig).

### ΔN/TAp63 mRNA expression ratio in cervical cancer and normal tissues

**Fig 2:**
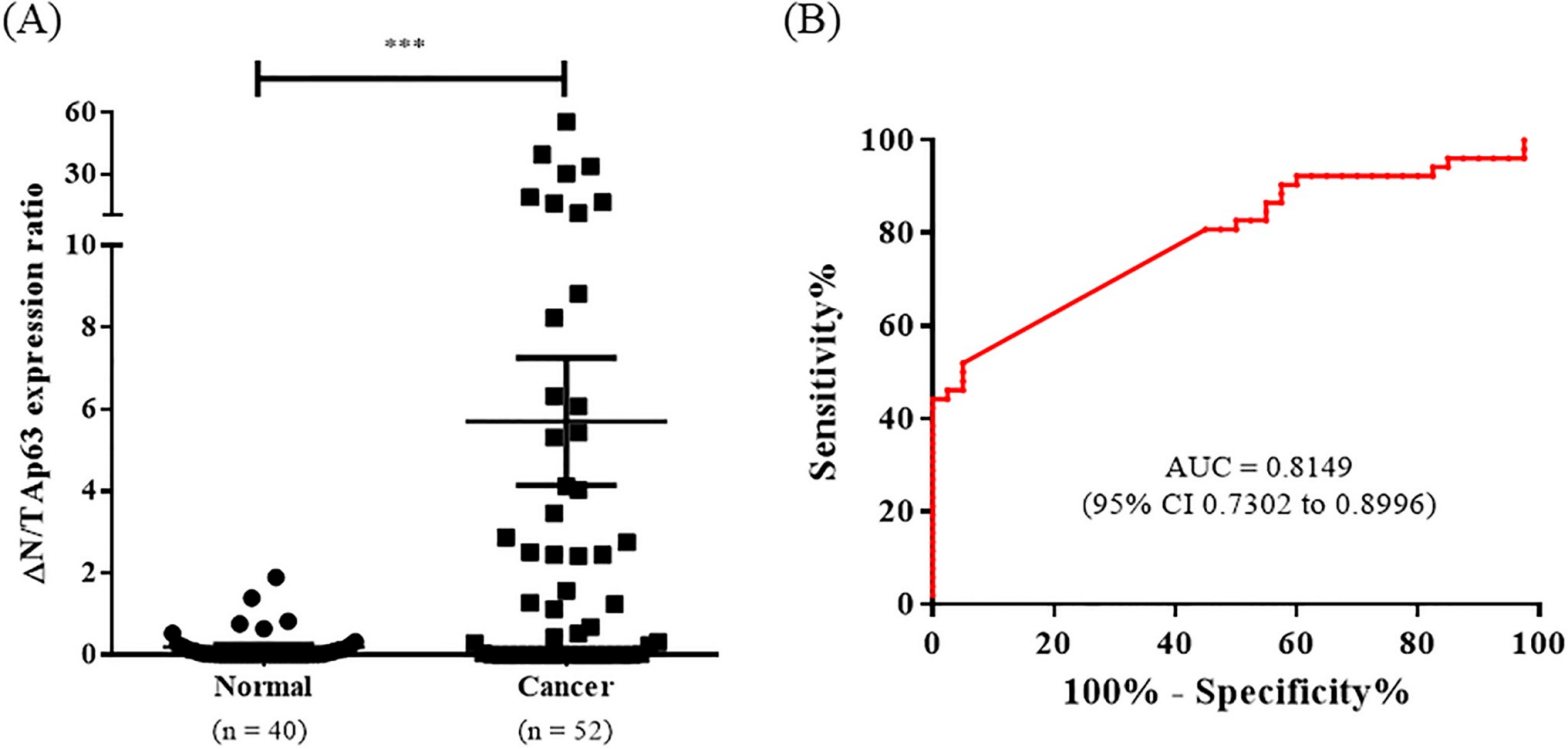
ΔN/TAp63 mRNA expression ratio of cervical cancer and normal FFPE tissues and ROC analysis. (A) The ΔN/TAp63 mRNA expression levels in 52 cervical cancer FFPE tissues and 40 normal FFPE tissues were measured by RT-qPCR. (B) ROC analysis showed that the AUC of the ΔN/TAp63 mRNA expression ratio was 0.8149. *P < 0.05, ** *P* < 0.01, ***P < 0.001.

To confirm that the disruption of the balance between TAp63 mRNA and ΔNp63 mRNA expression levels is associated with cervical cancer, the ΔN/TAp63 mRNA expression ratios in normal and cancer cervical tissues were analyzed. The mRNA expression ratio of ΔN/TAp63 was significantly higher in cancer tissues than in normal tissues (*P* = 0.003) (Fig 2A). The AUC of the ΔN/TAp63 mRNA expression ratio was clinically significant at 0.8149 (95% CI 0.7302–0.8996), which showed a better diagnostic value than that of ΔNp63 mRNA expression (Fig 2B). At a cut-off value of 1, the sensitivity of the ΔN/TAp63 mRNA expression ratio was 52% and the specificity was 95%. To identify whether the ΔN/TAp63 mRNA expression ratio was influenced by age group, we further analyzed the ΔN/TAp63 mRNA expression ratio by age in each cervical normal and cancer tissue. There was no significant difference of ΔN/TAp63 expression ratio according to age <50 years or >50 years in each cervical normal and cancer tissue (S2 Fig).

### ΔNp63 mRNA expression levels and ΔN/TAp63 mRNA expression ratio in precancerous LSIL and HSIL

**Fig 3:**
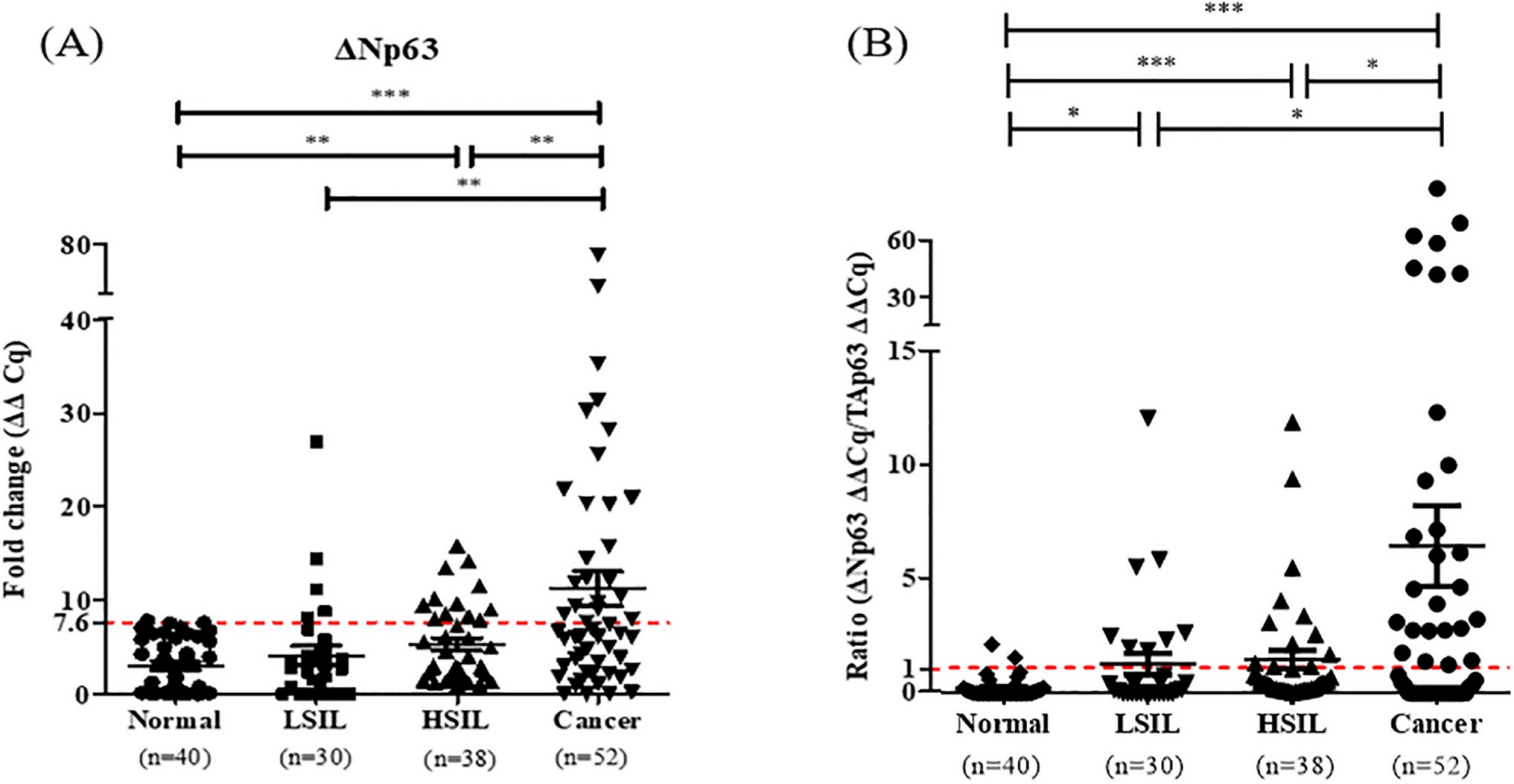
ΔNp63 expression levels and the ΔN/TAp63 expression ratio in normal, LSIL, HSIL, and cancerous tissues. (A) The ΔNp63 expression levels and (B) ΔN/TAp63 expression ratio in 40 normal, 30 LSIL, 38 HSIL, and 52 cancerous FFPE tissues were measured by RT-qPCR. *P < 0.05, **P < 0.01, ***P < 0.001.

ΔNp63 in the precancerous LSIL and HSIL was shown to have higher mRNA expression levels than that in normal tissues (*P* = 0.29 and *P* = 0.006), and lower mRNA expression levels in LSIL and HSIL than in cervical cancer tissues (*P* = 0.007 and *P* = 0.009) (Fig 3A). Additionally, at the cut-off value of 7.6 for ΔNp63, the ΔNp63 positive rate was 5.0% (2/40 cases) for normal, 16.7% (5/30 cases) for LSIL, 31.6% (12/38 cases) for HSIL, and 44.2% (23/52 cases) for cancerous tissues, which showed a gradual increase (Table 2).

**Table 2:** Association between ΔNp63 and ΔN/TAp63 expression ratio with cervical lesions.

ΔNp63 mRNA expression levels and ΔN/TAp63 expression ratio				
	ΔNp63-positive cases n (%)	*P*-value	Ratio-positive cases n (%)	*P*-value
Normal (n = 40)	2 (5.0)	Reference	2 (5.0)	Reference
LSIL (n = 30)^a^	5 (16.7)	0.1298	9 (30.0)	< 0.05
HSIL (n = 38)^b^	12 (31.6)	< 0.01	12 (31.6)	< 0.01
Cancer (n = 52)	23 (44.2)	< 0.001	27 (52.0)	< 0.001

^a^ LSIL, low-grade squamous intraepithelial lesions;

^b^ HSIL, high-grade squamous intraepithelial lesions.

ΔN/TAp63 in precancerous LSIL and HSIL was shown to have a higher mRNA expression levels than in normal tissues (*P* = 0.01 and *P* = 0.003) and lower mRNA expression levels in LSIL and HSIL than in cervical cancer tissues (*P* = 0.03 and *P* = 0.02) (Fig 3B). Additionally, at the cut-off value of 1 of the ΔN/TAp63 expression ratio, the positive rate was 5.0% (2/40 cases) for normal, 30.0% (9/30 cases) for LSIL, 31.6% (12/38 cases) for HSIL, and 52.0% (27/52 cases) for cancer tissues (Table 2). The ΔN/TAp63 mRNA expression ratio gradually increased from normal to LSIL, from LSIL to HSIL, and from HSIL to cancer tissues.

### Association ΔN/TAp63 mRNA expression ratio with tumor size in cervical cancer

**Fig 4:**
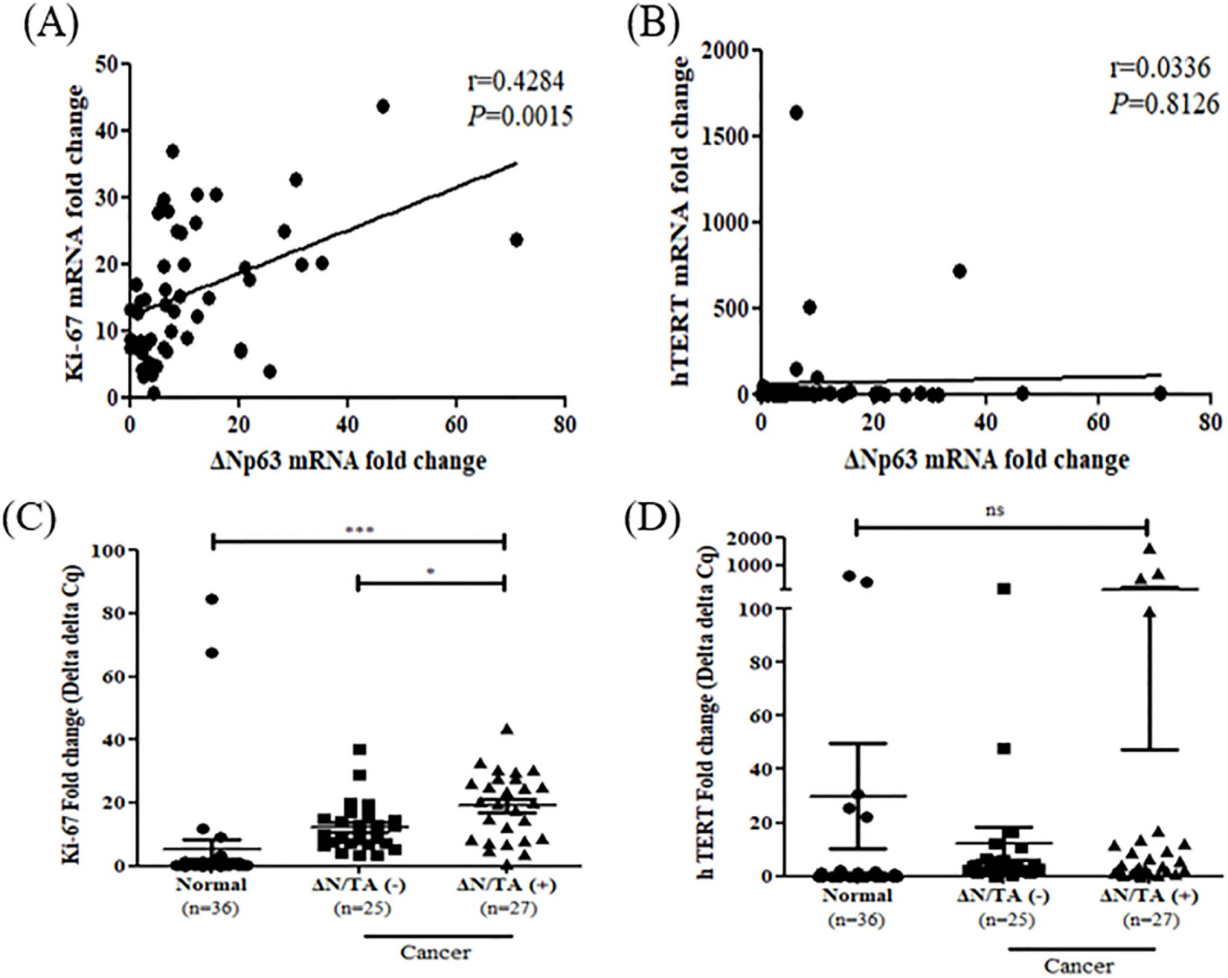
Correlation between ΔNp63 mRNA expression levels and the Ki-67 and hTERT mRNA expression levels and association of the Ki-67 and hTERT mRNA expression levels with the ΔN/TAp63 mRNA expression ratio. (A) Ki-67 and (B) hTERT mRNA expression levels were analyzed for their correlations with ΔNp63 mRNA expression levels using Pearson correlation (r = 0.4284, *P* = 0.0015 and r = 0.0336, *P* = 0.8126, respectively). (C) Ki-67 and (D) hTERT mRNA expression levels for the positive and negative groups for the ΔN/TAp63 mRNA expression ratio were analyzed using Student’s *t*-test. *P < 0.05, **P < 0.01, ***P < 0.001.

Among the clinicopathological parameters, the ΔN/TAp63 mRNA expression ratio was shown to have a positive correlation with bulky tumor size (*P* = 0.04) (Table 3). It was confirmed the association between the ΔN/TAp63 mRNA expression ratio and tumor size was link to the proliferation marker Ki-67, and the immortalization marker hTERT. Ki-67 mRNA expression levels were positively correlated with ΔNp63 mRNA expression levels (r = 0.43, *P* = 0.002), while hTERT mRNA expression levels were not correlated with ΔNp63 mRNA expression levels (r = 0.03, *P* = 0.81) (Fig 4A and 4B). Moreover, Ki-67 mRNA expression levels in the patients with positive ΔN/TAp63 mRNA expression ratio were significantly higher than in the patients with negative ΔN/TAp63 mRNA expression ratio (*P* = 0.02). No significant differences were found in hTERT mRNA expression levels according to the ΔN/TAp63 mRNA expression ratio (*P* = 0.15) (Fig 4C and 4D). These results suggested that the ΔNp63 mRNA and ΔN/TAp63 mRNA expression ratio was associated with cell proliferation.

**Table 3:** Association between ΔNp63 mRNA expression levels and ΔN/TAp63 mRNA expression ratio with clinicopathological parameters.

Variables	No. of cancer cases (n = 52)	ΔNp63 mRNA expression levels		*P*-value	ΔN/TAp63 expression ratio		*P*-value
		Positive (n)	Negative (n)		Positive (n)	Negative (n)	
**Age (year)**							
<50	19	9	10	0.11	8	11	0.39
≥50	33	14	19		19	14	
**Tumor size (cm)**							
<4	30	10	20	0.06	12	18	0.04
≥4	22	13	9		15	7	
**FIGO stage**^a^							
IA-IIA	21	11	10	0.51	11	10	0.93
IIB-IVB	28	12	16		15	13	
Unknown	3		3		1	2	
**Lymph node metastasis**							
No	28	11	17	0.45	13	15	0.57
Yes	22	11	11		12	10	
Unknown	2	1	1		2		
**HPV test**^b^							
Negative	8	3	5	1.00	4	4	1.00
Positive	44	20	24		23	21	

^a^FIGO, International Federation of Gynecology and Obstetrics;

^b^HPV, Human papillomavirus.

### Survival analysis between positive ΔNp63 mRNA and ΔN/TAp63 mRNA expression ratio

**Fig 5:**
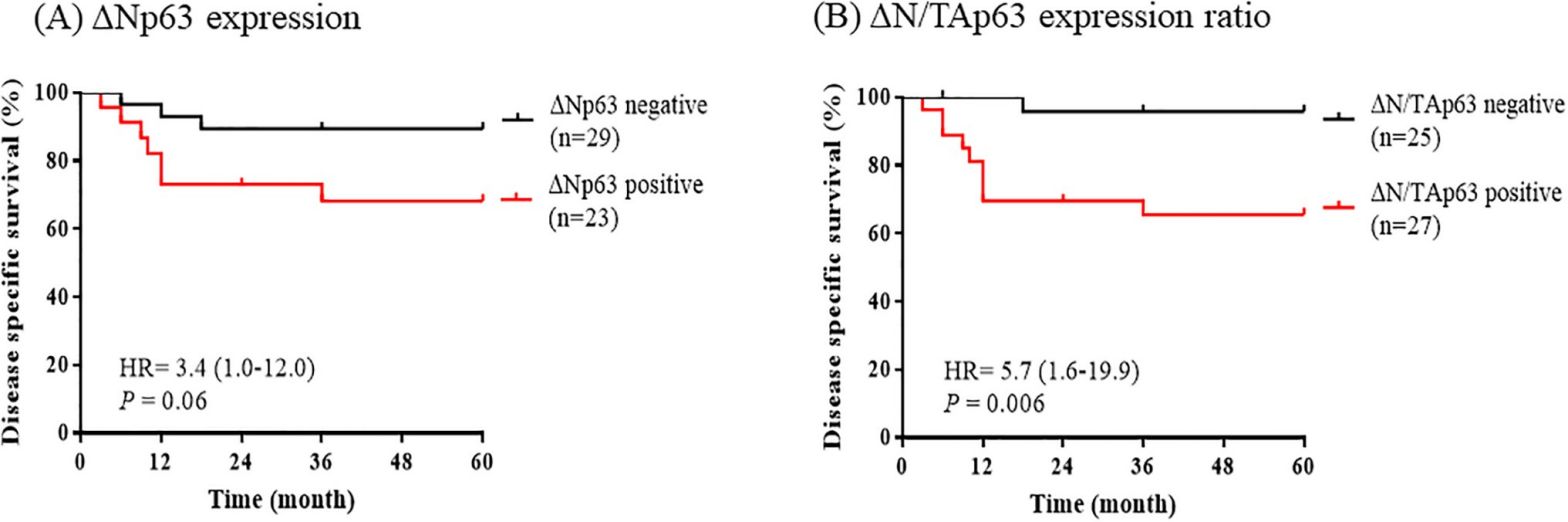
Survival analysis of ΔNp63 and ΔN/TAp63 mRNA expression ratio. Kaplan–Meier analysis in 52 cervical cancer patients were analyzed by log-rank test. (A) positive ΔNp63 mRNA expression (n = 23) and negative ΔNp63 mRNA expression (n = 29) were analyzed. (B) The positive ΔN/TAp63 mRNA expression ratio (n = 27) and negative ΔN/TAp63 mRNA expression ratio (n = 25) were analyzed. *P < 0.05, **P < 0.01, ***P < 0.001.

By Kaplan–Meier survival analysis, patients with positive ΔNp63 mRNA expression showed poor disease-specific survival than patients with negative ΔNp63 mRNA expression, but no statistically significant difference (HR = 3.4 [95% CI 1.0–12.0], *P* = 0.06). The survival rate in patients with positive ΔNp63 mRNA expression (n = 23) was 69.6%, and survival time ranged 35.6–54.5 months (mean, 45.0 months), whereas the survival rate in patients with negative ΔNp63 mRNA expression (n = 29) was 89.7%, and survival time ranged 49.5–60.4 months (mean, 54.9 months) (Fig 5A).

Notably, patients with positive ΔN/TAp63 mRNA expression ratio was shown to have significantly poorer prognoses than those with negative ΔN/TAp63 mRNA expression ratio (HR = 5.7 [95% CI 1.6–19.9], *P* = 0.006). The survival rate in patients with negative ΔN/TAp63 mRNA expression ratio (n = 25) was 96.0% and survival time ranged 54.9–61.6 months (mean, 58.3 months), whereas the survival rate in patients with positive ΔN/TAp63 mRNA expression ratio (n = 27) was 66.7% and survival time ranged 34.5–52.4 months (mean, 43.4 months) (Fig 5B).

## Discussion

The mortality rate in cervical cancer patients is approximately 50% worldwide and 30% in South Korea [18, 19]. To determine the tumor behavior and prognosis in each cervical cancer patient is important for improving cervical cancer prognosis and treatment strategies [18, 20, 21]. In this study, we aimed to investigate the characteristics of p63 in cervical cancer. p63 is known to be a prognostic marker in several cancers, but there are controversial characteristics (either tumor suppressive or oncogenic roles) of p63 isoforms.

The N-terminal of the p63 region, which has two promoters of p63 and encodes either TAp63 or ΔNp63 mRNA, was focused on in this study. To find out the two p63 isoforms, the mRNA expression levels of TAp63 and ΔNp63 in various cervical cancer cell lines ME-180, SiHa, CaSki, HeLa, and C33A were examined. The mRNA expression levels of ΔNp63 were significantly higher than those of TAp63 in ME-180, SiHa, CaSki, and C33A cells. Moreover, the mRNA expression levels of ΔNp63 in cervical cancer tissues were also significantly increased compared to those in cervical normal tissues. The mRNA expression levels of TAp63 were lower than those of ΔNp63, and were not significantly different between normal and cancerous cervical tissues. This result is concordant with the previous findings that the ΔNp63 to TAp63 mRNA expression ratio was higher in HeLa and Caski cells than those in the N-Tert and HaCaT normal cell lines [22].

TAp63 functions as a tumor suppressor, but some cervical cancer patients were shown to have a high expression of TAp63 mRNA in their cervical cancer tissue. From this result, we hypothesized whether not only the overexpression of ΔNp63, but also the imbalance of p63, is associated with cervical cancer carcinogenesis. Vakonaki et al. reported that an imbalance of the ΔNp63 and TAp63 isoforms is associated with grade I/II tumors in endometrial adenocarcinoma in a study of 20 paired adjacent normal and cancer tissues [23]. Therefore, we investigated the imbalance of p63, defined as the ΔN/TAp63 mRNA expression ratio, in cervical cancer cell lines and tissues.

The ΔN/TAp63 mRNA expression ratio in all five cervical cancer cell lines was shown to be overexpressed: ME 180 was the highest, followed by C33A, CaSki, SiHa, and HeLa. This indicates that the overexpression of ΔNp63 mRNA rather than TAp63 mRNA affected cervical cancer carcinogenesis. Moreover, the ΔN/TAp63 mRNA expression ratio in cervical cancer tissues was significantly higher than that in cervical normal tissues and was shown to have better sensitivity and diagnostic values compared to ΔNp63 mRNA.

Moreover, patients with positive ΔN/TAp63 mRNA expression ratio was shown to be significantly correlated with bulky tumor size and was higher expression of Ki-67, which is well known cell proliferation marker [24, 25]. Wagner et al. reported bulky tumor size in not only late stage but also early stage of cervical cancer was shown higher hazard ratio in a total of 18,649 cases [26]. The survival rate was significantly lower in patients with positive ΔN/TAp63 mRNA expression ratio than in those with negative. Therefore, we suggested that the ΔN/TAp63 mRNA expression ratio can be used as a prognostic marker for cervical cancer.

Because precancerous lesions are high risk to develop cervical cancer and the management is different from that of other cancers [27, 28], the patients diagnosed with precancerous LSIL and HSIL are needed to find out individual behavior in precancerous lesion. For these reason, p16/Ki67 dual staining in cervical precancerous lesions was assessed [29]. Our study showed that the ΔN/TAp63 mRNA expression ratio had a positive correlation with Ki67 and was also shown at high expression levels in LSIL and HSIL tissues compared to in normal cervical tissues. Therefore, the ΔN/TAp63 mRNA expression ratio can distinguish between normal, LSIL, HSIL, and cancerous tissues.

This study has a potential limitation of a small sample size of participants because of the single site of sample collection. A large-scale multi-center study to validate the ΔN/TAp63 mRNA expression ratio will be needed. Nevertheless, the ΔN/TAp63 mRNA expression ratio is a valuable concept to apply for clinical use as well as to understand the disruption of the ΔN/TAp63 mRNA expression ratio from *TP*63 gene in cervical cancer. A further study will be needed to compare the ΔN/TAp63 mRNA expression ratio with the data from immunofluorescence or immunohistochemistry assays.

## Conclusion

ΔNp63 mRNA and the ΔN/TAp63 mRNA expression ratio are useful for cervical cancer diagnosis. Especially, the ΔN/TAp63 mRNA expression ratio was found to be a potential poor prognostic and progression marker for cervical cancer. To clearly demonstrate the utility of these assays for cervical cancer, further large-scale studies of cervical cancer clinical data and samples are necessary.

## Supporting information

S1 Fig TAp63 and ΔNp63 mRNA expression levels of cervical cancer and normal FFPE tissues and receiver operating characteristics (ROC) analysis. (A) The TAp63 and (B) ΔNp63 mRNA expression levels in 52 cervical cancer FFPE tissues and 40 normal FFPE tissues were measured by RT-qPCR. ROC analysis showed that the AUC of (C) TAp63 mRNA was 0.5135 and that of (D) ΔNp63 was 0.7529. *P < 0.05, **P < 0.01, ***P < 0.001.
(TIF)

S2 Fig ΔN/TAp63 mRNA expression ratio in cervical normal and cervical FFPE tissues according to age groups. (A) The ΔN/TAp63 mRNA expression ratio in 40 cervical normal FFPE tissues and 52 cervical cancer FFPE tissues were analyzed by age group with a cutoff at 50 years in study participants. No significant differences in the ΔN/TAp63 mRNA expression ratio were evident between the two age groups in cervical normal and cancer FFPE tissues (*P* = 0.56 and *P* = 0.95). n.s. not statistically significant.
(TIF)

S1 Table Oligonucleotide primers used in this study. (XLSX)
