# Correction: ΔNp63 to TAp63 expression ratio as a potential molecular marker for cervical cancer prognosis

**DOI:** 10.1371/journal.pone.0216968

**Published:** 2019-05-09

**Authors:** Sunyoung Park, Suji Lee, Jungho Kim, Geehyuk Kim, Kwang Hwa Park, Tae Ue Kim, Dawn Chung, Hyeyoung Lee

## Notice of republication

An incomplete, earlier version of this article was published in error. This article was republished on May 1, 2019 to correct for this error. Please download the article again to view the correct version.
