# Supplementary figures and images for: ΔNp63 to TAp63 expression ratio as a potential molecular marker for cervical cancer prognosis
Source: PLoS One. 2019 Apr 11;14(4):e0214867. doi: 10.1371/journal.pone.0214867 (PMC6459502; doi:10.1371/journal.pone.0214867)

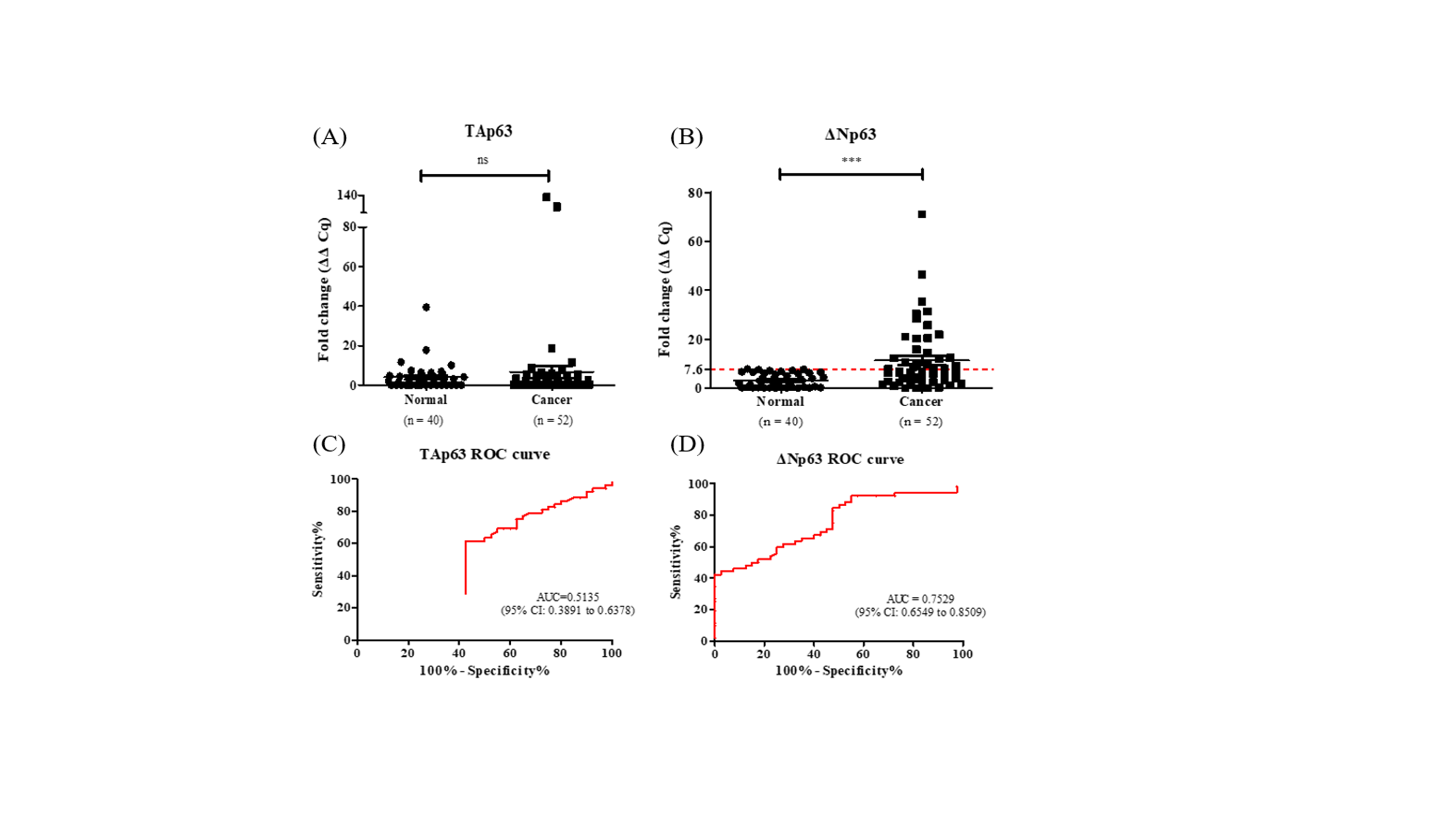

Supplement: S1 Fig — (A) The TAp63 and (B) ΔNp63 mRNA expression levels in 52 cervical cancer FFPE tissues and 40 normal FFPE tissues were measured by RT-qPCR. ROC analysis showed that the AUC of (C) TAp63 mRNA was 0.5135 and that of (D) ΔNp63 was 0.7529. *P < 0.05, **P < 0.01, ***P < 0.001. (TIF) [file pone.0214867.s001.tif]

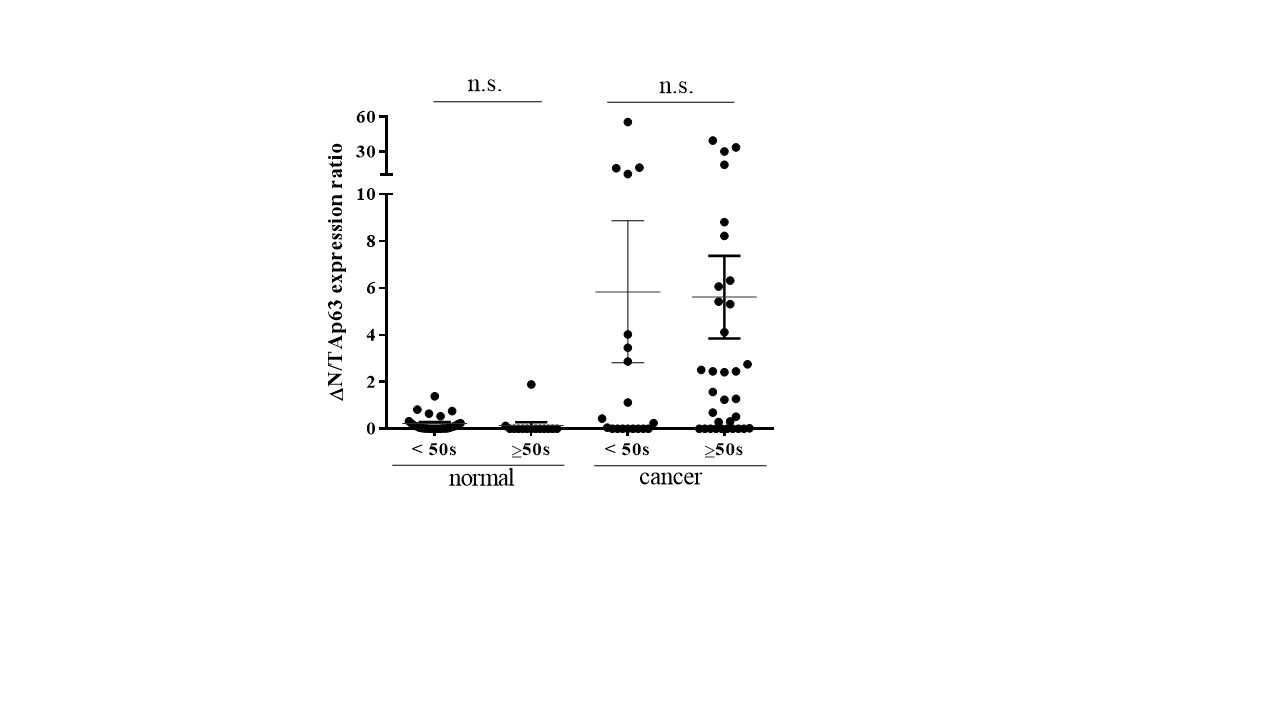

Supplement: S2 Fig — (A) The ΔN/TAp63 mRNA expression ratio in 40 cervical normal FFPE tissues and 52 cervical cancer FFPE tissues were analyzed by age group with a cutoff at 50 years in study participants. No significant differences in the ΔN/TAp63 mRNA expression ratio were evident between the two age groups in cervical normal and cancer FFPE tissues (P = 0.56 and P = 0.95). n.s. not statistically significant. (TIF) [file pone.0214867.s002.tif]
